# Effectiveness of Cranberry Capsules to Prevent Urinary Tract Infections in Vulnerable Older Persons: A Double-Blind Randomized Placebo-Controlled Trial in Long-Term Care Facilities

**DOI:** 10.1111/jgs.12593

**Published:** 2014-01-17

**Authors:** Monique A A Caljouw, Wilbert B van den Hout, Hein Putter, Wilco P Achterberg, Herman J M Cools, Jacobijn Gussekloo

**Affiliations:** * Department of Public Health and Primary Care, Leiden University Medical CenterLeiden, the Netherlands; † Department of Medical Decision Making, Leiden University Medical CenterLeiden, the Netherlands; ‡ Department of Medical Statistics, Leiden University Medical CenterLeiden, the Netherlands

**Keywords:** geriatrics, long-term care facility, urinary tract infection, prevention, cranberry

## Abstract

To determine whether cranberry capsules prevent urinary tract infection (UTI) in long-term care facility (LTCF) residents.

**Design:**

Double-blind randomized placebo-controlled multicenter trial.

**Setting:**

Long-term care facilities (LTCFs).

**Participants:**

LTCF residents (N = 928; 703 women, median age 84).

**Measurements:**

Cranberry and placebo capsules were taken twice daily for 12 months. Participants were stratified according to UTI risk (risk factors included long-term catheterization, diabetes mellitus, ≥1 UTI in preceding year). Main outcomes were incidence of UTI according to a clinical definition and a strict definition.

**Results:**

In participants with high UTI risk at baseline (n = 516), the incidence of clinically defined UTI was lower with cranberry capsules than with placebo (62.8 vs 84.8 per 100 person-years at risk, *P *=* *.04); the treatment effect was 0.74 (95% confidence interval (CI) = 0.57–0.97). For the strict definition, the treatment effect was 1.02 (95% CI = 0.68–1.55). No difference in UTI incidence between cranberry and placebo was found in participants with low UTI risk (n = 412).

**Conclusion:**

In LTCF residents with high UTI risk at baseline, taking cranberry capsules twice daily reduces the incidence of clinically defined UTI, although it does not reduce the incidence of strictly defined UTI. No difference in incidence of UTI was found in residents with low UTI risk.

Urinary tract infection (UTI) is a common bacterial infection in residents of long-term care facilities (LTCF),^1^,^2^ accounting for nearly 25% of all infections.^3^,^4^ UTI not only causes several days of illness, but may have more-severe consequences such as delirium, dehydration, urosepsis, hospitalization, or even death.^5^,^6^

Interventions to prevent UTI could reduce these severe consequences,^7^ but there are no evidence-based interventions that decrease UTI in institutionalized populations.^1^ The use of prophylactic antibiotics is currently controversial because of side-effects and antibiotic resistance.^8^,^9^ Prophylaxis with cranberry is a potential prevention strategy.^10^,^11^ Cranberries contain proanthocyanidins (PACs), which are stable phenolic compounds with anti-adhesion activity against *Escherichia coli*.^12^–^14^ In vitro, antibacterial activity of concentrated cranberry juice against other pathogens such *Staphylococcus aureus*, *Pseudomonas aeruginosa*, *Klebsiella pneumoniae*, and *Proteus mirabilis* has also been demonstrated.^15^,^16^

There is aggregated evidence that cranberry juice may lead to a decrease in the incidence of symptomatic UTIs over a 12-month period, particularly in women with recurrent UTIs.^17^,^18^ Another recent systematic review indicates that cranberry-containing products are associated with a protective effect against UTI in different subgroups, albeit with heterogeneity across the included trials.^19^ A recent study in children without urological abnormalities showed a 65% reduction of UTI with the use of cranberry.^20^

Two studies reported that cranberry juice may be protective in subgroups of older adults,^21^,^22^ but the effectiveness of cranberry capsules in the protection against UTI in vulnerable older persons in LTCFs has not been studied.

The present study assessed the effectiveness of cranberry capsules in preventing UTI in vulnerable older persons living in LTCFs. Research in an institutionalized population is challenging, and clinical manifestations of UTI may be subtle.^23^–^25^ To be relevant for clinical practice and science, a clinical definition according to international guidelines for LTCF residents and a strict definition according to scientific criteria were both used.

## Methods

### Design

This was a double-blind randomized placebo-controlled multicenter trial in two strata, based on baseline UTI risk. Twenty-one LTCF organizations from the University Nursing Home Research Network in South Holland, the Netherlands, participated.

The medical ethics committee of the Leiden University Medical Center approved the study. Written informed consent was obtained from all participants. A guardian provided written consent for participants incapable of giving informed consent because of cognitive impairment.

### Study Participants

LTCF residents aged 65 and older were included. Exclusion criteria were use of coumarin and a life expectancy of 1 month or less. Coumarin users were excluded because of a possible interaction between coumarin and cranberry, leading to higher international normalized ratios and bleeding.^26^–^28^

After informed consent and before randomization, medical records were studied to stratify participants according to baseline UTI risk. Participants with long-term catheterization (>1 month), diabetes mellitus, or at least one UTI in the preceding year were considered to be at high UTI risk.

Within two strata of UTI risk, participants were randomized into the cranberry or placebo group. Block randomization (blocks of 6) was used, stratified for risk profile and ability to give informed consent, generated using a computer random number generator. Participants, family, nursing staff, physicians, pharmacists, and research nurses were blinded to treatment, and the random numbers were put in sealed envelopes so the research nurse could allocate to the treatment group (cranberry or placebo) directly on the ward. Only the supplier of the capsules knew the codes given to the capsules (cranberry or placebo).

### Intervention

Participants were randomly assigned to take cranberry or placebo capsules twice daily for 12 months. Participants already using a cranberry supplement stopped using their own cranberry products before randomization and changed to the study capsule at baseline. The cranberry capsules contain 500 mg of the product, with 1.8% proanthocyanidins (9 mg). The placebo was indistinguishable in color, taste, and appearance, consisting of cellulose microcrystal colored red with azorubin.

The physician prescribed the coded capsules, and the pharmacist added them to the drug-dispensing systems. Nurses distributed the capsules and recorded whether the participant took them on a drug kardex. Adherence was measured over 1 month by counting all capsules that the participants took during the fifth month of intervention and comparing that with the prescribed number of capsules.

### Outcome Measures

The primary outcome was incidence of UTI. There is no criterion standard in diagnosing UTI in LTCF residents. Most clinical criteria to ascertain UTI are based on consensus.^29^–^31^ A recent study showed that micturition-related signs and symptoms are predictive of UTI.^32^

Because of the absence of a criterion standard in the study population, this study used a clinical definition and a strict UTI definition. The clinical definition of UTI is a broad and practical definition following clinical practice guidelines for LTCF residents.^24^,^25^ This clinical definition of UTI is based on the presence of a minimum of one of the following characteristics: specific and nonspecific micturition-related symptoms and signs, a positive test (nitrite test, leukocyte esterase test, dipslide, or culture), antibiotic treatment for UTI, or UTI reported in the medical record.

Specific symptoms and signs are pain before, during, or after micturition; increased frequency of micturition; pain in abdomen; hematuria; foul smell; and signs of common sickness (fever >37.9°C or 1.5°C above baseline temperature, chills, nausea, vomiting). Nonspecific symptoms are anorexia, fatigue and reduced mobility, and signs of delirium (e.g., confusion, deterioration in mental or functional status).

The strict UTI definition is based on a scientific approach, including the presence of micturition-related symptoms and signs confirmed with a positive dipslide or culture. A urine dipslide or culture was considered to be positive when there were 10^5^ CFU/mL or more bacteria, with no more than two species of organisms present.

The treating physicians diagnosed the UTI and reported the presence of UTI in the medical record. For this study, they reported the needed study information on a prestructured case report form, including presence of specific and nonspecific micturition-related signs and symptoms, kind of testing and results, and antibiotic treatment. Secondary outcomes were incidence of recurrent UTI, hospitalization, and mortality.

In a companion cost-effectiveness article, whether the effectiveness of cranberry capsule use is attained at reasonable costs was investigated.^33^

At 6 and 12 months, a research nurse visited all participants in their LTCF to check their medical records for the occurrence of UTIs and to verify that all UTIs were collected during the study period. Side effects and reasons for withdrawal from the study were registered.

### Additional Measurements at Baseline

A research nurse interviewed all participants at baseline in their LTCF, where face-to-face interviews were conducted. If participants were not able to answer, their responsible nurse was interviewed. Information on participant sociodemographic characteristics and medical history were obtained at baseline. Care dependency was assessed using the Care Dependency Scale (CDS),^34^ which measures 15 items of basic care needs on an aggregate scale from 15 to 75.

### Sample Size

Sample size was based on an expected incidence of 44 first UTIs per 100 residents per year in the placebo group. To demonstrate a 40% reduction in incidence of UTI with the use of cranberries,^22^ 500 residents needed to be included in each stratum (2 strata of 2 groups of 250)—1,000 residents in total (dropout rate 10%, alpha 0.05, power 80%).

### Statistical Analysis

Differences in baseline characteristics between treatment groups were compared using the Student *t*-test for continuous variables and chi-square for categorical variables. The incidence of UTI was calculated using the life-table method. The number of first UTIs was assigned to the numerator and the number of observed person-years at risk was assigned to the denominator. The observed person-years at risk were counted from randomization to end of study, to date of death, or to date of first UTI. The cumulative incidence of UTI for cranberry and placebo was calculated accounting for mortality as competing risk.^35^ The difference in the cumulative incidence of UTI in residents between cranberry and placebo was tested using the log-rank test. The treatment effect of cranberry with respect to placebo was investigated using Cox proportional hazards models, expressed as hazard ratios (HRs).

The number needed to treat (NNT) was calculated over 1 year of follow-up, based on the difference in proportion of being event free in the placebo and cranberry group.^36^,^37^ The difference in NNT between treatment groups was tested using a *z*-test; *P *≤* *.05 was considered significant.

To investigate possible heterogeneity in UTI rates between individuals, a gamma-frailty model was fitted,^38^ a random effect model for time-to-event data in which the random effect (frailty) has a multiplicative effect on baseline hazard function. Analyses were performed based on intention to treat using SPSS for Windows version 17.0 (SPSS Inc., Chicago, IL) and R version 2.13.0 (R Foundation for Statistical Computing, Vienna, Austria).

## Results

**Figure 1:**
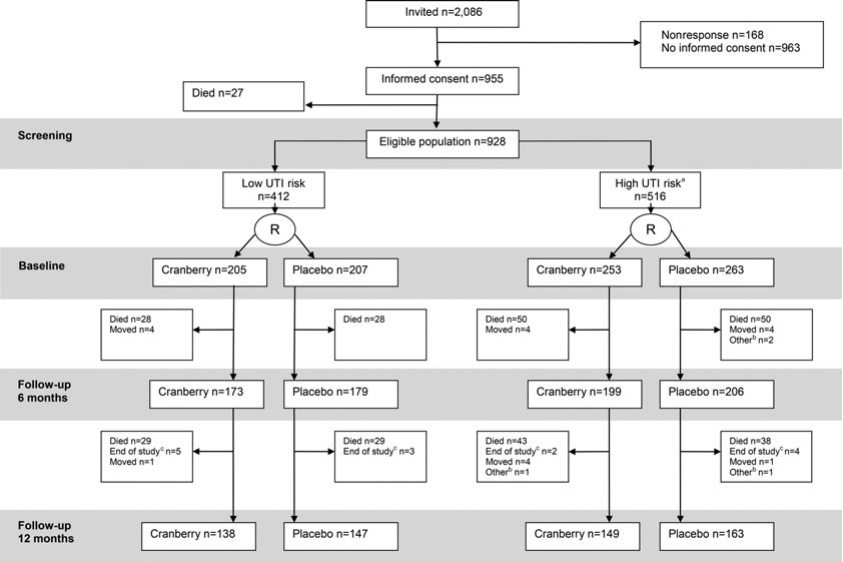
Participant recruitment and follow-up. ^a^Diabetes mellitus or urinary catheter or treated urinary tract infection in past 12 months. ^b^No adherence, withdrawn by elderly care physician or family. ^c^Intervention stopped because of end of the study period. UTI = urinary tract infection.

Between November 2008 and August 2009, all 2,086 eligible residents were invited to participate in a letter, and then a research nurse orally invited them. The study stopped in June 2011. Twenty-seven of the 955 residents who gave written informed consent died before randomization, resulting in a study population of 928 participants (Figure1). A nonresponder analysis for giving informed consent showed no difference between nonresponders and responders in age, sex, or UTI risk profile. None of the participants had end-stage renal disease.

Four hundred twelve low-UTI-risk and 516 high-UTI risk-participants were included. There were no baseline differences within the UTI risk groups between the cranberry and placebo groups (Table1). Mean capsule intake was 97% (95% confidence interval (CI) = 96.6–97.6%) and was similar between the randomization groups and between the risk groups.

**Table 1:** Baseline Characteristics of Study Population at Randomization (Intention-to-Treat Analysis)

Characteristic	Low UTI Risk, n = 412		High UTI Risk, n = 516*	
	Cranberry, n = 205	Placebo, n = 207	Cranberry, n = 253	Placebo, n = 263
Sociodemographic				
Female, n/N (%)	143/205 (69.8)	159/207 (76.8)	188/253 (74.3)	213/263 (81.0)
Age, n, median (IQR)	205, 84.0 (78.5–88.5)	207, 83.0 (79.0–88.0)	253, 85.0 (79.0–89.0)	263, 84.0 (79.0–88.0)
Length of stay, months, n, median (IQR)	204, 18.0 (4.0–42.0)	205, 19.0 (4.0–39.0)	251, 17.0 (6.0–41.0)	263, 19.0 (6.0–39.0)
Family informed consent, n/N (%)	180/205 (87.8)	185/207 (89.4)	205/253 (81.0)	212/263 (80.6)
Functioning				
15-item Care Dependency Scale score (range 15–75), n, median (IQR)	199, 42.0 (30.0–56.0)	197, 45.0 (30.5–55.0)	244, 44.0 (31.0–56.0)	250, 43.0 (30.0–56.0)
Cranberry use before start of study, n/N (%)	3/196 (1.5)	6/202 (3.0)	18/248 (7.3)	22/253 (8.7)
Urinary incontinence, n/N (%)	138/198 (69.7)	136/199 (68.3)	152/247 (61.5)	157/246 (63.8)
Urinary catheter, n/N (%)	0/205 (0.0)	0/207 (0.0)	49/253 (19.4)	47/263 (17.9)
Infections in past 12 months				
Urinary tract infection, n/N (%)	0/205 (0.0)	0/207 (0.0)	203/253 (80.2)	200/263 (76.0)
Number of UTIs in past 12 months, n, median (IQR)	–	–	202, 1.0 (1.0–2.0)	199, 2.0 (1.0–2.0)
Antibiotics for UTI suppression, n/N (%)	0/196 (0.0)	1/202 (0.5)	3/248 (1.2)	5/253 (2.0)
Lower respiratory tract infection, n/N (%)	35/200 (17.5)	35/204 (17.2)	47/249 (18.9)	54/259 (20.8)
Other infection, n/N (%)	21/200 (10.5)	24/204 (11.8)	38/248 (15.3)	33/255 (12.9)
Comorbidities, n/N (%)				
Renal dysfunction	20/201 (10.0)	16/206 (7.8)	37/252 (14.7)	34/262 (13.0)
Urogenital surgery	40/200 (20.0)	45/203 (22.2)	50/253 (19.8)	66/262 (25.2)
Myocardial infarction	14/203 (6.9)	17/205 (8.3)	25/252 (9.9)	25/262 (9.5)
Stroke	38/204 (18.6)	33/205 (16.1)	64/251 (25.5)	76/261 (29.1)
Cancer	38/202 (18.8)	42/203 (20.7)	42/252 (16.7)	48/259 (18.5)
Diabetes mellitus	0/205 (0.0)	0/207 (0.0)	79/253 (31.2)	103/263 (39.2)
Chronic obstructive pulmonary disease	36/203 (17.7)	26/199 (13.1)	40/250 (16.0)	32/256 (12.5)
Dementia	162/199 (81.4)	177/207 (85.5)	174/250 (69.6)	187/261 (71.6)

IQR = interquartile range; UTI = urinary tract infection.

* Diabetes mellitus or urinary catheter or treated urinary tract infection in past 12 months.

### Incidence of UTI

**Figure 2:**
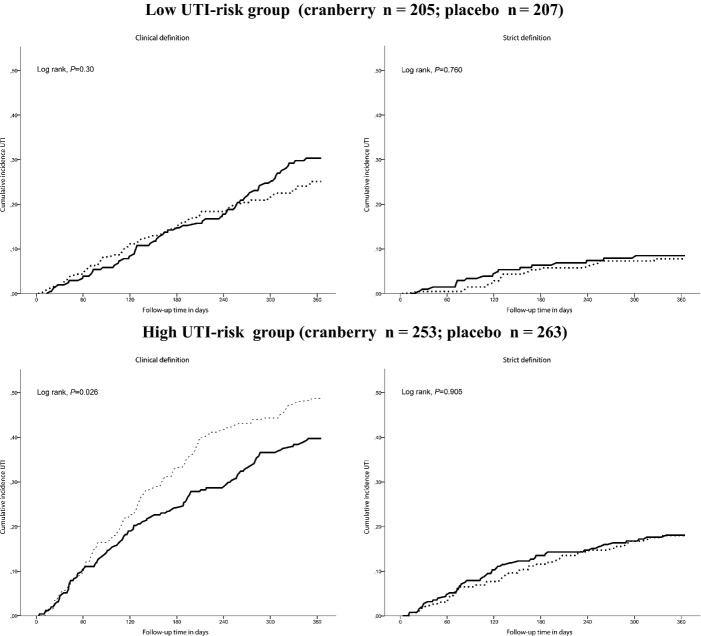
Cumulative incidence of urinary tract infection (UTI) within 1 year of follow-up depending on treatment (cranberry or placebo) stratified for those at low and high UTI risk, accounting for mortality as competing risk. Solid line: cranberry group; dotted line: placebo group. Clinical definition: symptom or positive testing (nitrite test, leukocyte esterase test, dipslide or culture) or antibiotic treatment or UTI reported in the medical record. Strict definition: symptom and positive dipslide or culture.

In the high-UTI-risk group, the curve of cumulative incidence of clinically defined UTI showed a positive treatment effect from 2 months of follow-up onward (*P *=* *.03). No such effect was found for strictly defined UTI (*P *=* *.91). There was no difference between cranberry and placebo in the low-UTI-risk group (Figure2).

In the high-UTI-risk group, the incidence of UTI according to the clinical definition was 62.8 per 100 person-years at risk (95% CI = 50.3–75.2) for cranberry and 84.8 per 100 person-years at risk (95% CI = 70.0–99.7) for placebo (*P *=* *.04). The treatment effect in those at high UTI risk was 0.74 (95% CI = 0.57–0.97). The incidence for UTI following the strict definition was not different in those using cranberry and placebo. The treatment effect was 1.02 (95% CI = 0.68–1.55; Table2). A subanalysis in participants without long-term catheters in the high-UTI-risk group (n = 420) showed a larger treatment effect of cranberry capsules than of placebo for clinically defined UTI (Table2). According to the clinical definition, five high-risk residents need to be treated with cranberry for 1 year to prevent one resident free of UTI for 1 year (*P *=* *.01).

**Table 2:** Incidence of Urinary Tract Infection (UTI), According to Two Definitions, Depending on Treatment with Cranberry for Different Definitions and UTI Risk During 12 Months of Follow-Up

UTI Risk	Events, n		Person-Days at Risk		Incidence Per 100 Person Years at Risk (95% CI)		Risk Difference (95% CI)	Treatment Effect, Hazard Ratio (95% CI)	*P-*Value
	Cranberry	Placebo	Cranberry	Placebo	Cranberry	Placebo			
Low^a^									
Clinical^b^	59	51	53,498	55,806	40.3 (30.0–50.5)	33.4 (24.2–42.5)	6.9 (−6.9–20.7)	1.22 (0.84–1.77)	.30
Strict^c^	17	16	58,888	61,812	10.5 (5.5–15.5)	9.4 (4.8–14.1)	1.1 (−5.7–7.9)	1.11 (0.56–2.20)	.76
High^d^									
Clinical	98	125	56,989	53,783	62.8 (50.3–75.2)	84.8 (70.0–99.7)	−22.0 &#6(−(41.4 to −2.7)	0.74 (0.57–0.97)	.03
Strict	45	46	64,888	68,248	25.3 (17.9–32.7)	24.6 (17.5–31.7)	0.7 (−9.5–11.0)	1.02 (0.68–1.55)	.91
High without long-term catheter^e^									
Clinical	71	99	47,569	44,382	54.5 (41.8–67.2)	81.4 (65.4–97.5)	−26.9 (−47.4 to −6.5)	0.67 (0.49–0.91)	.01
Strict	31	39	53,585	55,775	21.1 (13.7–28.5)	25.5 (17.5–33.5)	−4.4 (−15.3–6.5)	0.83 (0.52–1.33)	.43

CI, confidence interval.

a Cranberry, n = 205; placebo, n = 207.

b Symptom or positive testing (nitrite test, leukocyte esterase test, dipslide or culture) or antibiotic treatment or UTI reported in the medical record.

c Symptom and positive dipslide or culture.

d Cranberry, n = 253; placebo, n = 263.

e Cranberry, n = 204; placebo, n = 216.

In the low-UTI-risk group, the incidence of UTI according to the clinical definition was 40.3 per 100 person-years at risk (95% CI = 30.0–50.5) for cranberry and 33.4 per 100 person-years at risk (95% CI = 24.2–42.5) for placebo (*P *=* *.30).

### Recurrent UTI

In a gamma-frailty model (a random effect model) using all recurrent clinical UTIs, cranberry did not significantly reduce the UTI rate in the high-UTI-risk group (HR = 0.92, 95% CI = 0.71–1.17, frailty variance 0.62, *P *<* *.001). In the low-UTI-risk group, the HR of cranberry versus placebo was 1.14 (95% CI = 0.78–1.68, frailty variance 1.50, *P *<* *.001).

### Hospitalization and Mortality

Five participants (0.5%), all from the high-UTI-risk group, were hospitalized during follow-up for UTI, with no difference between cranberry and placebo (*P *=* *.62). In the low-UTI-risk group, 114 (27.7%) participants died during follow-up, of whom three died from UTI (cranberry vs placebo *P *=* *.56). In the high-UTI-risk group, 181 participants (35.1%) died during follow-up, of whom 14 (7.7%) died from UTI, with no difference between cranberry and placebo (7 vs 7 *P *=* *.91).

## Discussion

This double-blind randomized placebo-controlled multicenter trial investigated the effectiveness of cranberry capsules to prevent UTI in older LTCF residents. In participants with high UTI risk, twice-daily intake of cranberry capsules resulted in a 26% lower incidence of clinically defined UTI than placebo, but no difference was found in UTI incidence of strictly defined UTI. In residents with low UTI risk, twice-daily intake of cranberry capsules did not result in a lower incidence of UTI than with placebo.

### Effectiveness

A systematic review showed that cranberry-containing products were associated with a protective effect against UTI in certain populations.^19^ A Cochrane review reported a UTI reduction of 35% (95% CI = 10–54%).^17^ In the recent update of this Cochrane review in 2012, the authors performed a meta-analysis based on two studies evaluating cranberry in elderly adults (N = 413).^18^ Cranberry did not significantly reduce UTI in this population (risk ratio = 0.75, 95% CI = 0.39–1.44).^18^ In contrast with this last review, the current study found a positive effect of treatment with cranberry capsules on the incidence of clinically diagnosed UTI in 516 older persons with high UTI risk. A possible explanation for this difference could be the product used (juice vs capsules), study population (hospitalized vs institutionalized), and sample size. Another study comparing cranberry with low-dose trimethoprim (follow-up 6 months) showed no difference between cranberry and low-dose antibiotics but did not include a placebo arm.^39^

It could have been expected that the beneficial effect of cranberry capsules would be fairly prompt after starting treatment, but the current study showed a beneficial effect of cranberry capsules in the high-UTI-risk group starting from 2 months of treatment on for clinically defined UTI. This was shown in an earlier study that found a reduction that started between 1 and 2 months after initiating cranberry juice and remained stable throughout the 6 months of follow-up.^21^ Cranberries with PAC were expected to have an effect by different mechanisms, because they influence the adhesive capacity of fimbriae of bacteria and build a biofilm on the surface, preventing adhesion. Nevertheless, bacteria could be persistent (chronic bacteriuria), and the types of bacteria could vary over time. So cranberry protects against UTI, but it takes some time to have an effect.

Because the effect of preventive care depends on the incidence of the disease, a preplanned stratification was made at baseline on baseline UTI risk. Based on the literature, LTCF residents with diabetes mellitus,^40^–^42^ long-term catheterization,^24^,^43^,^44^ or UTI in the preceding year^3^,^42^,^45^,^46^ were considered to be at high risk. Although a group of LTCF residents with a high incidence were selected using these criteria, it might be that other criteria would have selected a group with even higher risks or more preventable UTIs.

### Strengths and Limitations

This is the first large study of the effectiveness of cranberry capsules in preventing UTI in LTCF residents. These residents represent a vulnerable older population, with a median age of 85 and older, severely dependent on care, high infection rates, high levels of comorbidity, and 1-year mortality of 35%^47^—a population in which clinical manifestations of UTI may be subtle.^23^

The current study was performed in Dutch LTCFs—intramural care settings where elderly care physicians provided medical care.^48^–^53^ Medication prescription and distribution are well organized. The study capsules were added to the existing drug-dispensing system. Medication distribution by nurses is routine in Dutch LTCFs, and participants rarely missed taking any capsule, which was reflected in a high adherence rate, although adherence was assessed only in the fifth month, which might not be generalizable to the other months. This high and similar level of adherence in both treatment groups also suggests that capsules were well tolerated, side-effects were negligible, and blinding remained adequate during the study.

A technical assessment of blinding was not performed, although the research nurses did not receive information about deblinding during the study visits. In addition, the distribution and adherence of participants in the cranberry and placebo groups were similar.

The clinical definition for UTI is based on a broad definition of UTI (symptoms and signs, positive test, antibiotic treatment, or reported in the medical record) and relies on the clinical judgment of the elderly care physician, in accordance with international clinical guidelines for UTIs in LTCFs.^24^,^25^ Although this UTI definition is different from the strict definition, it reflects clinical care in LTCFs and adds knowledge to practice guidelines to assist physicians in making decisions.

The strict definition of UTI is based on a scientific approach, including micturition-related symptoms and signs confirmed according to a positive culture or dipslide, and could be used for comparisons with studies in other populations but is difficult to generalize to clinical practice in LTCFs. It is generally accepted that diagnosing UTI in vulnerable older persons, especially in long-term care, is complicated. A recent study in nursing home residents with advanced dementia showed that symptoms and signs of UTI are frequently not present in older persons with dementia.^51^ In the current study, for example, most participants had dementia (76%) or incontinence (64%). Therefore, a clean catch urine sample for culturing is often not available, making it impossible to diagnose UTI according to the strictest criteria.^52^ The current study was double-blinded, so the randomization itself cannot have influenced the clinical definition. Despite the large study sample, no treatment effect was shown in those at high UTI risk using the strict definition. The study may have been slightly underpowered for the rarer strictly defined UTIs.

The optimum dosage of cranberries is not clear, and a well-designed dose-finding study is needed. An in vitro study suggests that the administration of 72 mg of PAC daily may offer some protection against bacterial adhesion in the bladder.^53^ The daily use of 18 mg of PAC (two capsules) in this study may not have been high enough.

These results are not automatically generalizable to vulnerable older persons living at home. Differences not only in vulnerability and infection rates, but also in adherence and hospitalization rates were expected.

## Conclusions

In LTCF residents with high UTI risk, taking cranberry capsules twice daily results in a 26% lower incidence of clinically defined UTI than with placebo, although no difference was found in UTI incidence according to a strict definition. Cranberry capsules may offer an opportunity to decrease the incidence of this common infection in high-UTI-risk LTCF residents by using a well-tolerated treatment.
